# Fluorescence measurements show stronger cold inhibition of photosynthetic light reactions in Scots pine compared to Norway spruce as well as during spring compared to autumn

**DOI:** 10.3389/fpls.2014.00264

**Published:** 2014-06-13

**Authors:** Tapio Linkosalo, Juha Heikkinen, Pertti Pulkkinen, Raisa Mäkipää

**Affiliations:** Vantaa Unit, Finnish Forest Research InstituteVantaa, Finland

**Keywords:** Scots pine, Norway spruce, photosynthesis, phenology, cold inhibition, climate change, frost tolerance

## Abstract

We studied the photosynthetic activity of Scots pine (*Pinus sylvestris* L.) and Norway spruce (*Picea abies* [L.] Karst) in relation to air temperature changes from March 2013 to February 2014. We measured the chlorophyll fluorescence of approximately 50 trees of each species growing in southern Finland. Fluorescence was measured 1–3 times per week. We began by measuring shoots present in late winter (i.e., March 2013) before including new shoots once they started to elongate in spring. By July, when the spring shoots had achieved similar fluorescence levels to the older ones, we proceeded to measure the new shoots only. We analyzed the data by fitting a sigmoidal model containing four parameters to link sliding averages of temperature and fluorescence. A parameter defining the temperature range over which predicted fluorescence increased most rapidly was the most informative with in describing temperature dependence of fluorescence. The model generated similar fluorescence patterns for both species, but differences were observed for critical temperature and needle age. Down regulation of the light reaction was stronger in spring than in autumn. Pine showed more conservative control of the photosynthetic light reactions, which were activated later in spring and more readily attenuated in autumn. Under the assumption of a close correlation of fluorescence and photosynthesis, spruce should therefore benefit more than pine from the increased photosynthetic potential during warmer springs, but be more likely to suffer frost damage with a sudden cooling following a warm period. The winter of 2013–2014 was unusually mild and similar to future conditions predicted by global climate models. During the mild winter, the activity of photosynthetic light reactions of both conifers, especially spruce, remained high. Because light levels during winter are too low for photosynthesis, this activity may translate to a net carbon loss due to respiration.

## Introduction

Trees growing in the boreal zone must respond to a wide range of climatic conditions during a typical year. For evergreens, this means that photosynthetic organs must be active in the summer but become dormant and frost-resistant for the winter (Leinonen and Hänninen, 2002). Spring is a demanding time for boreal conifers when light is abundant but cold air temperatures and possibly lack of available water can hinder photosynthesis (Suni et al., 2003). Trees are locally adapted to prevailing conditions, but global warming will change the synchronization of temperature and light regime at a given location, i.e., although temperatures are expected to be higher, incident radiation will remain the same. Increasing temperatures will affect respiration during autumn and winter (Vesala et al., 2010) and possibly enhance photosynthetic activity during spring (Hall et al., 2009). Therefore, climate change will impact the productivity of boreal forests (Wang et al., 2011), but the nature of that impact will depend on how trees adapt to the new temperature-light regime (TLR).

The photosynthetic pathway in coniferous trees is typical and consists of two parts; a light reaction captures photons and stores their energy in short-lived compounds before a dark (i.e., light independent) reaction converts them into more stable products. The efficiency of the light reaction is largely determined by incident radiation while the dark reaction is a complex biochemical process more dependent on prevailing temperatures (Porcar-Castell et al., 2008). Consequently, a cold but bright TLR could lead to unchecked light reactions producing free radicals that damage plant tissues (Huner et al., 1998; Porcar-Castell et al., 2008). In response to this problem, boreal evergreens can actively downgrade light reactions taking place in their photosynthetic tissues (Kolari et al., 2007).

As expected for locally-adapted species, the potential photosynthesis of evergreens follows local changes in the TLR (Ottander and Öquist, 1991; Beck et al., 2004). Due to the asynchrony of temperature and light, the phenology of a given species will affect its photosynthesis, growth, reproduction, and risk of frost damage in altered climates of the future. Development of the photosynthetic capacity of boreal conifer species has been extensively studied (Bergh et al., 1998; Lundmark et al., 1998; Leinonen and Hänninen, 2002; Mäkelä et al., 2004; Kolari et al., 2007), but comparative studies with different species in the same environmental conditions are limited (Lundmark et al., 1988; Ögren et al., 1997). Clearly, if conifers show species-specific responses to a given change in TLR, consequent changes to the productivity of boreal forests in the future will be determined by the co-distribution of species and the new climate. Comparative analyses of species living in similar conditions and modeling the data appropriately would improve the precision with which impacts of climate change on productivity and ecosystem health can be predicted. However, observation periods must include a wide range of climatic conditions in order to detect and investigate any differences among species.

The goal of this study was to understand the light reaction dynamics of Norway spruce and Scots pine in response to temperature variation during the year. We also compared chlorophyll fluorescence between spring and autumn. We hypothesized that in comparison to Scots pine, photosynthetic light reactions in Norway spruce resume earlier in spring and remain at higher levels for most of the winter. We fitted nonlinear mixed-effect models to light reaction activity based on average and minimum daily temperatures, and compared the predictions for spruce and pine in relation to changes in TLR.

## Materials and methods

We measured the development of fluorescence in 51 Norway spruce (*Picea abies* [L.] Karst) and 48 Scots pine (*Pinus sylvestris* L.) located in the Haapastensyrjä common garden (Finnish Forest Research Institute) in southern Finland (60°37′, 24°25′, 1250 d.d.). Most sample trees originate from Finland (60–70°N) but some of the spruce trees came from populations in central Europe (Table 1). Norway spruce are growing in a stand planted in 1980 and were approximately 15 m tall when measurements were taken. For practical reasons, data were collected from shoots that were on the outer edges of the spruce stand where trees had healthy shoots at least down to breast-height level. We assumed this to be an indication that these shoots received sufficient light to maintain good condition.

**Table 1:**
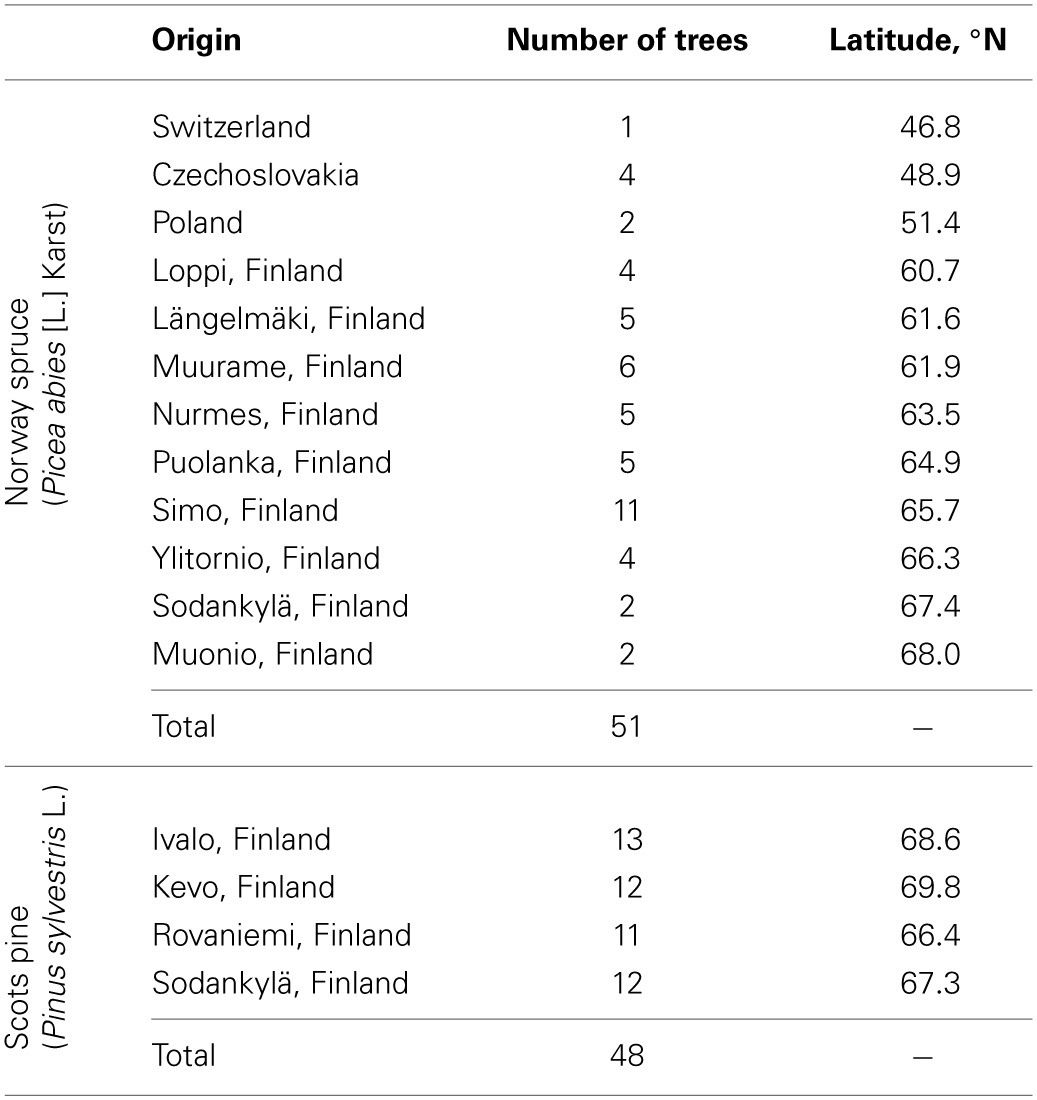
**Origin and number of trees studied in this experiment**.

The Scots pine were grafted from shoots collected from mature trees in spring 2006 to rootstocks sown a year before. The grafts were planted in an open field adjacent to the spruce stand in 2007. In 2013, the trees were about 1.5–2.0 m tall; data were collected from the upper branches. All Scots pine originated from northern Finland (Table 1).

Fluorescence measurements began 06 March 2013 and continued until the end of February 2014. Measurements were generally made weekly, increasing to 2–3 times a week when abrupt weather changes and consecutive changes in the fluorescent activity of the trees were expected. Measurements began with needles formed during 2012 (hereafter referred to as 2012 needles), and were later expanded to include needles that formed in the early summer of 2013 (hereafter 2013 needles), once they had bursted. Both 2012 and 2013 needles were measured in parallel until their fluorescence was the same in early July, after which only 2013 needles were measured. Measuring needles of different ages enabled the development of fluorescence between spring (i.e., 2012 needles) and autumn (i.e., 2013 needles) to be observed. Fluorescence was measured in the field with a hand-held Pocket PEA fluorimeter (Hansatech, Ltd.), where needles were first enclosed in light-proof clips for at least 30 mins to allow all photosynthetic reactions time to complete. The fluorimeter was then attached to the clip and a strong pulse of light was passed through the sample. Initially, most of the light energy is absorbed and processed by the dark reaction and the fluorescent irradiance is at its lowest value (*F*_*o*_). Shortly thereafter the dark reaction becomes saturated and surplus energy from the light reaction causes the fluorescent irradiance to reach a peak value (*F*_*m*_). The fluorimeter records the initial and saturated fluorescent irradiance and calculates a relative index as:

(1) $$\frac{F_{m}\;-\;F_{O}}{F_{m}}=\frac{F_{v}}{F_{\text{m}}}$$

The expression *F*_*v*_/*F*_*m*_ reflects the photosynthetic potential: when light reactions are suspended, there is minimal change in the fluorescent irradiance and, therefore, values are small, while high values indicate that the photosynthetic apparatus is active.

**Figure 1:**
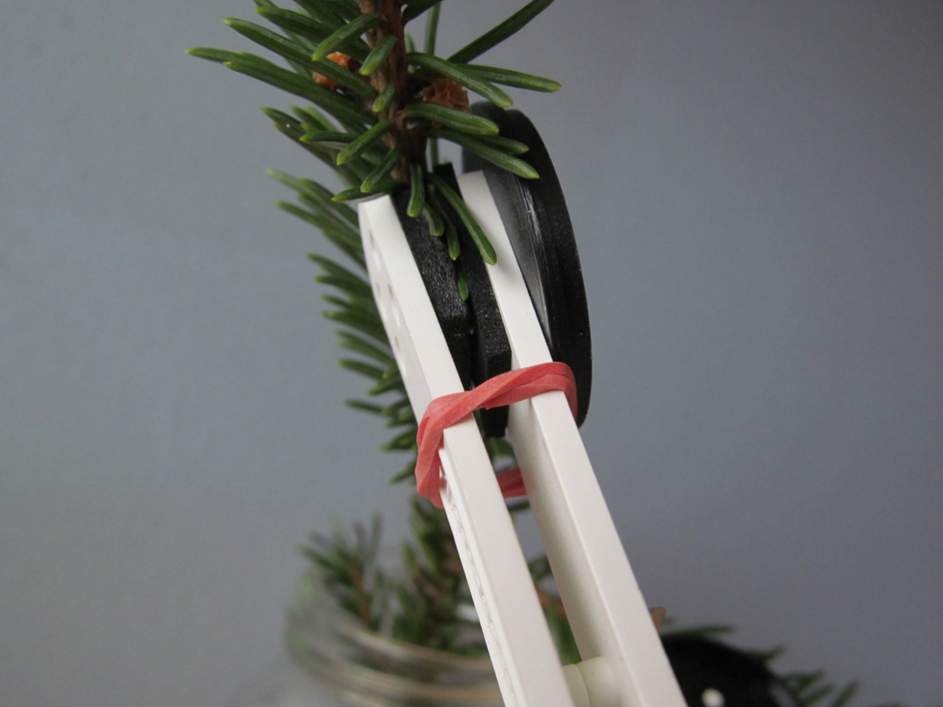
**Clips used to measure chlorophyll fluorescence modified with an extra foam pad added to the lower jaw and a small rubber band to draw the clip closed**. These modifications helped provide a light-proof closure of the clip around the needles to be measured.

Leaf clips for the Pocket PEA fluorimeter are intended to measure broadleaf species with flat leaves. We modified the clips to ensure a light-proof closure of the clip around the cylindrical sample needles (Figure 1). For each tree, one shoot was selected and tagged for measurement. Typically, two needles were inserted into the aperture of the clip. In repeated measurements, placement of the clip on the shoot was selected randomly and independently of earlier samples.

**Figure 2:**
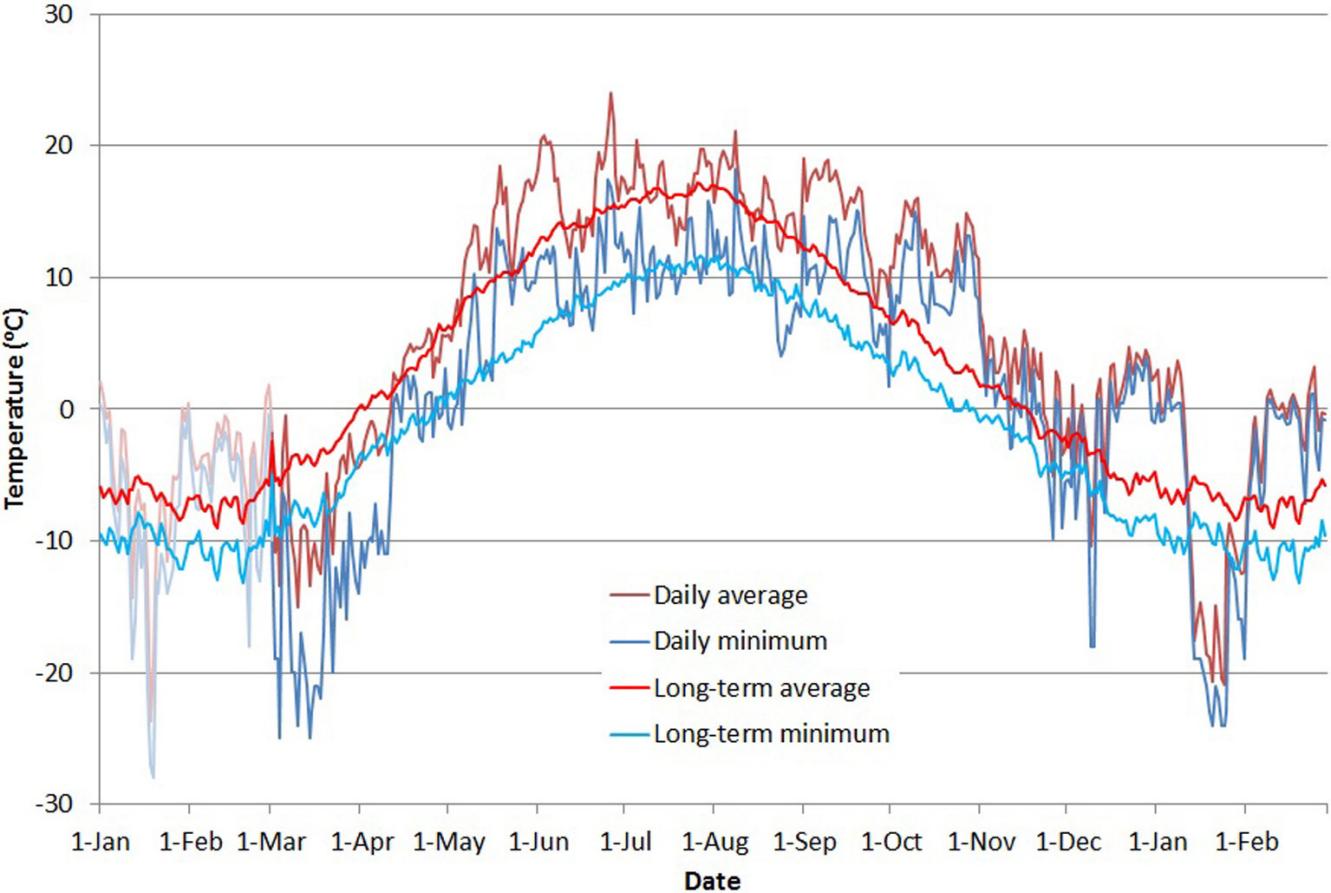
**Average (red line) and minimum (blue line) daily temperatures during the study period**. Stronger colors indicate the period of fluorescence measurements, with 2 months preceding the period presented in shaded colors. The thinner red and blue lines show the long-term mean and minimum daily temperatures (red and blue, respectively) from the Finnish Meteorological Institute weather data from the years 1961 to 2012 (Venäläinen et al., 2005).

Throughout the measuring period, outdoor temperatures were recorded with a NTC-type thermistor located in a weather-logger box in an open field about 200 m from the stands. Temperature was measured once per minute. Daily average temperature was calculated as the arithmetic average of all daily measurements, and daily minimum temperature was produced by selecting the minimum value for each day (Figure 2). To account for the time lag in the reaction of trees to changing conditions, we calculated a moving average of the temperature using a range of time constants from 1 to 12 days based on values reported in the literature (see discussion). For comparison (Figure 2), we calculated a long-term daily average for 1961–2012 from temperature measurements provided by the Finnish Meteorological Institute (Venäläinen et al., 2005).

The dependence of fluorescence measurements (photosynthetic activity) on temperature was described with a non-linear mixed effects model based on a four-parameter logistic response function:

(2) $$F=a_{\text{min}}+\;\frac{a_{\text{max}}\;-\;a_{\text{min}}}{1\;+\text{ exp ​}\left[\frac{T\;-\;c}{b}\right]},$$

where *F* is the predicted fluorescence, *T* is the sliding average of either the daily average or daily minimum temperature, *a*_*min*_ and *a*_*max*_ are the minimum and maximum asymptotes, respectively, *b* is a scale parameter determining the rate of ascent (i.e., a lower value of *b* translates to a steeper rate of ascent), and *c* is the temperature at which *F* is halfway between *a*_*min*_ and *a*_*max*_. This temperature value also corresponds to the largest change of fluorescence activity. Variable *c* is referred to as the crossover temperature, interpreted as the temperature value characterizing the critical temperature range for photosynthetic light reactions. The asymptotic values *a*_*min*_ and *a*_*max*_ were fitted as fixed parameters separately for each species. The other two parameters, *b* and *c*, were fitted separately for each species as well as for the set of observations representing spring and autumn (i.e., 2012 and 2013 needles). Furthermore, *b* and *c* were allowed to vary between trees so that the value of *b* for the *i*th observation is as follows:

(3) $$b_{i}=b_{s\left(i\right),\;y(i)}+\beta_{i},$$

where *s*(*i*) and *y*(*i*) indicate the species and needle cohort, respectively, and β_*i*_ is a zero-mean tree-specific random effect; *c*_*i*_ was modeled in a similar fashion. Variances for the random effect were estimated separately for each species, but a common estimate for residual variance was appropriate. Based on the latter assumption, both species could be included in the same model, allowing for the statistical testing of differences between them. Model fitting was carried out in the R-environment (R Core Team, 2013) with the nlme package (Pinheiro et al., 2013), which uses approximate maximum likelihood (Lindstrom and Bates, 1990). For Norway spruce, association between tree origin and cross-over temperature was assessed with Spearman's rank correlation (Best and Roberts, 1975) of latitude of origin and estimated tree-specific random effect in *c_i_*.

## Results

**Figure 3:**
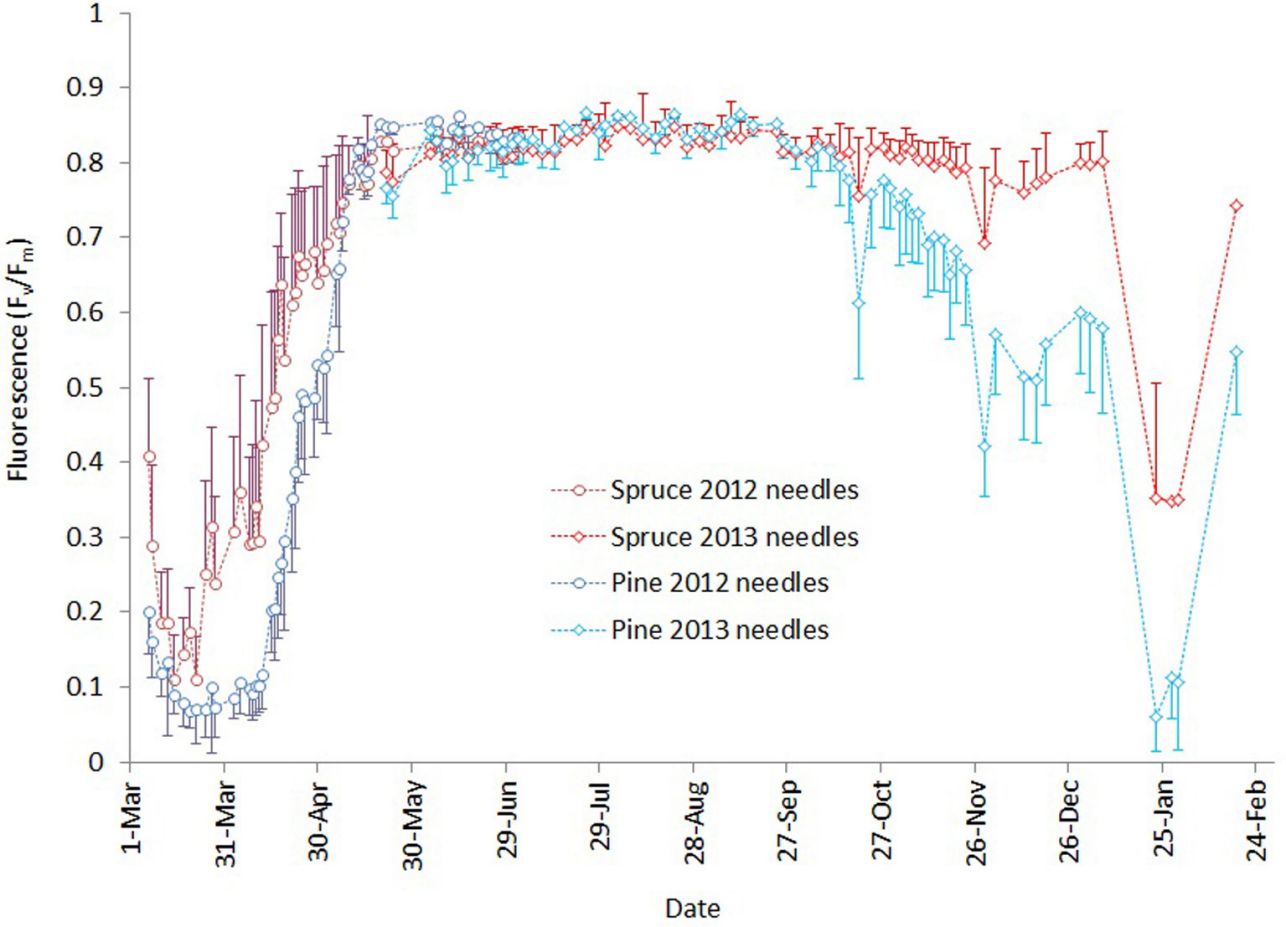
**Development of chlorophyll fluorescence March 2013–February 2014**. Darker blue and red lines with circles indicate measurements for needles that burst in spring 2012 for pine and spruce, respectively. Brighter lines with diamonds are the corresponding values for needles that burst in spring 2013. Error bars show the standard deviation of measurements for each day.

Fluorescence values (Figure 3) showed that in spring 2013 the activity of the photosynthetic light reactions in Norway spruce started to develop earlier, (i.e., at lower air temperatures) than in Scots pine. Fluorescence in Scots pine was lower than that of spruce throughout the 6-week period of development of photosynthesis in the spring. Both pine reached the same fluorescence level as spruce by the end of May.

During the autumn, Scots pine began attenuating light reactions earlier and to lower levels compared to Norway spruce, but as long as autumn temperatures remained mild, fluorescence activity did not reach the lowest levels observed in the previous spring. The winter of 2013–2014 was unusually mild in southern Finland, with only mildly freezing temperatures except for a cold period of 3 days in early December and another of 2 weeks in late January (Figure 1). During the first cold spell, pine fluorescence decreased to a much lower level than spruce. In both species, the photosynthetic apparatus was reactivated after the cold spell, but pine resumed at a lower level than spruce. The cold period in January 2014 caused pine fluorescence to decrease to a similar level to the lowest observed in early spring 2013, while spruce persisted at a higher level. Light reactions of both species reactivated when air temperatures warmed, but pine resumed activity at a lower level compared to spruce.

A range of time constant values for the moving average of temperature was tested, and values were compared by fitting the mixed model to each temperature record. The log-likelihood values of the model for each value of the time constant are given in Table 2. A time constant value of 7 days gave the largest log-likelihood, but differences among adjacent values were small. Also, daily mean or daily minimum temperature only had a slight effect on fit of the mixed model (Table 2).

**Table 2:** **The log-likelihood values of the mixed model with different time constant values for the moving mean and minimum daily temperatures**.

**Time constant**	**Log-likelihood**	
**Days**	**Mean temperature**	**Minimum temperature**
1	12.771	9428
2	14.029	11.476
3	14.359	12.546
4	14.545	13.483
5	14.648	14.094
6	14.692	14.339
7	14.695	14.432
8	14.669	14.457
9	14.621	14.439
10	14.559	14.393
11	14.486	14.326
12	14.407	14.243

**Figure 4:**
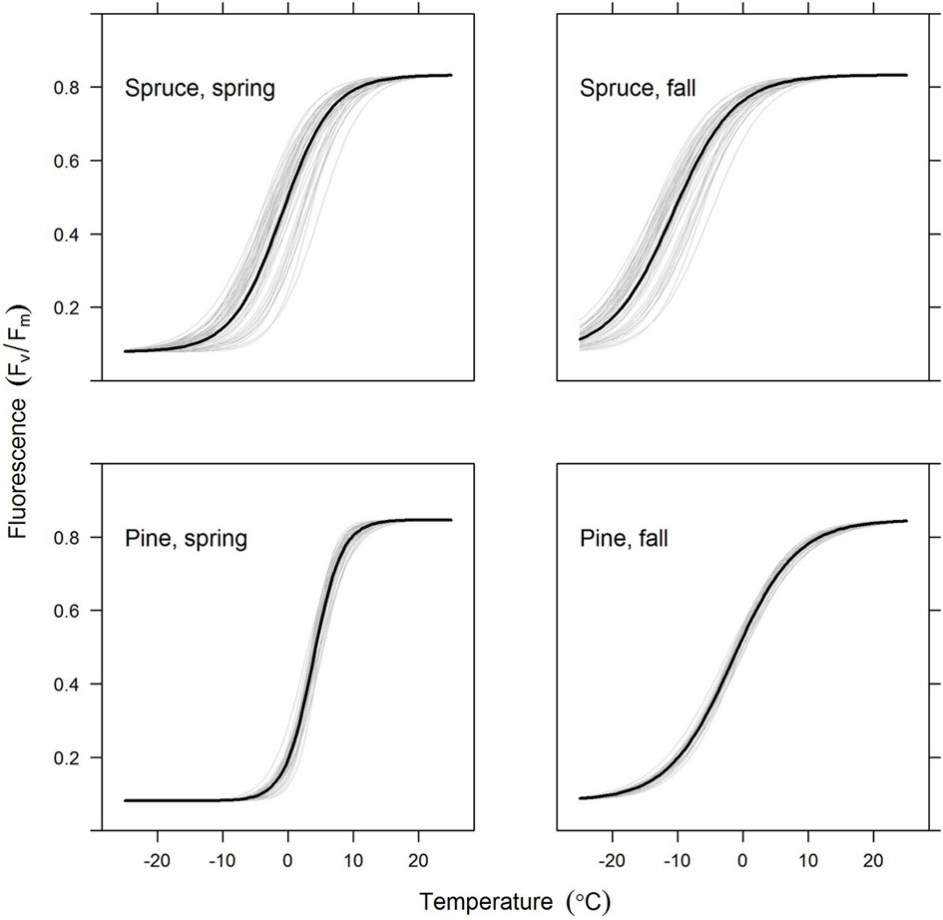
**Predicted chlorophyll fluorescence (*F*_*v*_/*F*_*m*_) as a function of temperature for the spruce and pine needles (upper and lower rows, respectively) and for the old and new needles (left and right columns, respectively)**. The thick lines show the average model for all trees in the group, with thin lines indicating individual trees. Figures are based on the best-fitting model using a 7-day sliding average of daily mean temperature.

The crossover temperature parameter value, which indicates the temperature where the change of fluorescent activity is at its largest, was lower for spruce compared to pine and also lower for spring needles (or “2012 needles”) compared to autumn needles (“2013 needles”) (Table 3, Figure 4). This means that for any given temperature below the saturating temperature, the photosynthetic activity of spruce is higher than that of pine. Needles were more active in autumn than during spring for both species. The slope of fluorescence development was steeper for pine in the spring. This suggests a more rapid development of photosynthetic capacity as temperatures increase, which compensates for some of the effect of the higher crossover temperature. According to the fitted models, differences in all other parameter values except for *a*_*min*_ between species and needle origins are clearly statistically significant (*p*-values < 0.0001).

**Table 3:**
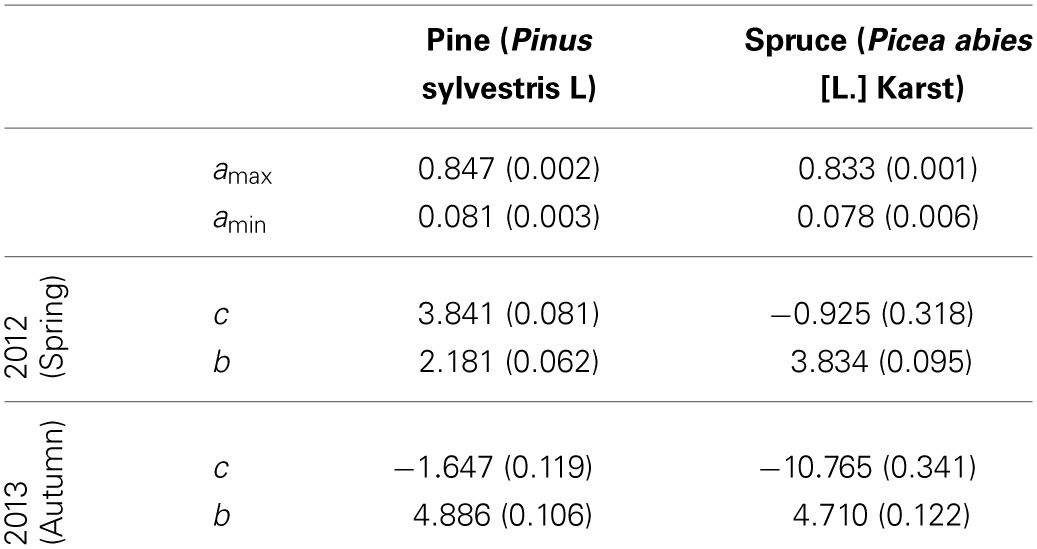
**Parameter estimates and standard errors (in parentheses) for the fixed effects in the non-linear mixed model using a 7-day sliding average of daily mean temperature as a predictor**.

**Figure 5:**
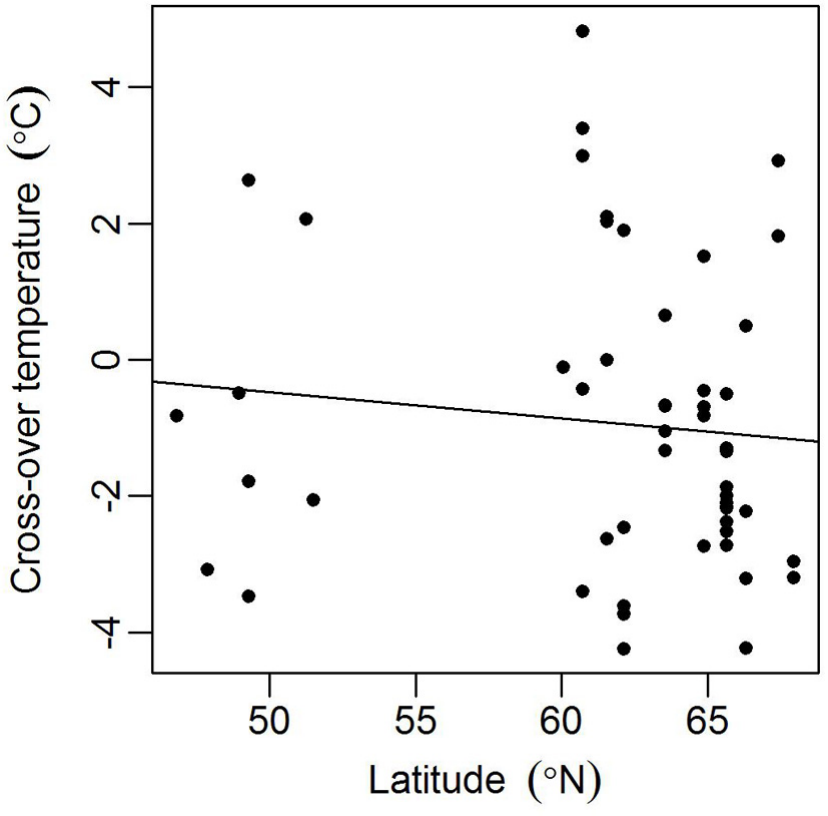
**Association between the latitude of origin and estimated tree-specific cross-over temperature *c*_*i*_ for spruces in the spring (2012 needles)**. Spearman's rank correlation ρ = −0.23 (*p* = 0.10).

Models for individual trees show greater variation for spruce than pine (Table 3, Figure 4). Spruce trees were more variable in terms of their origin, being drawn from throughout Finland (i.e., 60–66°N) and locations in central Europe, while pines all came from northern Finland (Table 1). However, association between the latitude of origin and cross-over temperature was not statistically significant in our data (Figure 5).

## Discussion

Our measurements of chlorophyll fluorescence indicate that the activity of photosynthetic light reactions of boreal conifers follows ambient air temperature. In spring 2013, *F*_*v*_/*F*_*m*_ reached minimum levels in March after a cold period of several weeks. Air temperatures late in 2013 and during January–February 2014 were unusually warm, up to 6°C above the long-term average (Figure 1). During this period, *F*_*v*_/*F*_*m*_ values remained high, with spruce almost reaching levels observed during the summer (Figure 3). Studies of net ecosystem exchange using eddy-covariance methods (Van Dijk and Dolman, 2004; Vesala et al., 2010) have shown that trees maintain their respiration during such warm but dark periods, which suggests that photosynthetic dark reactions are also active during warm spells in winter. The low *F*_*v*_/*F*_*m*_ values observed after the cold period in January 2014 indicate a down-regulation of light reactions. Örlander (1993) suggested that this down-regulation is due to damage to photosystem II caused by light reception in subfreezing temperatures. However, the Norway spruce and Scots pine in our common garden experiment quickly recovered from this decline, and the *F*_*v*_/*F*_*m*_ ratio followed the temperature increase. This indicates that photosystem II of the conifers can sustain freeze-thaw cycles of at least –20°C, with rapid recovery during milder periods. Norway spruce tended to maintain a higher *F*_*v*_/*F*_*m*_ ratio, and reactivated as rapidly as Scots pine, which showed stronger photoinhibition during cold spells. Differences in the activity of the photosynthetic apparatus can be linked to the way these two conifers down-regulate respiration. Ögren et al. (1997) showed that cold tolerance of conifers is correlated with the soluble sugar content of their needles and that Norway spruce is more frost tolerant than Scots pine or Lodgepole pine—a proposal that is in conflict with results presented here. The authors also observed that spruce down-regulated respiration faster and more extensively than pine, which improved the conservation of sugar storage in spruce.

**Figure 6:**
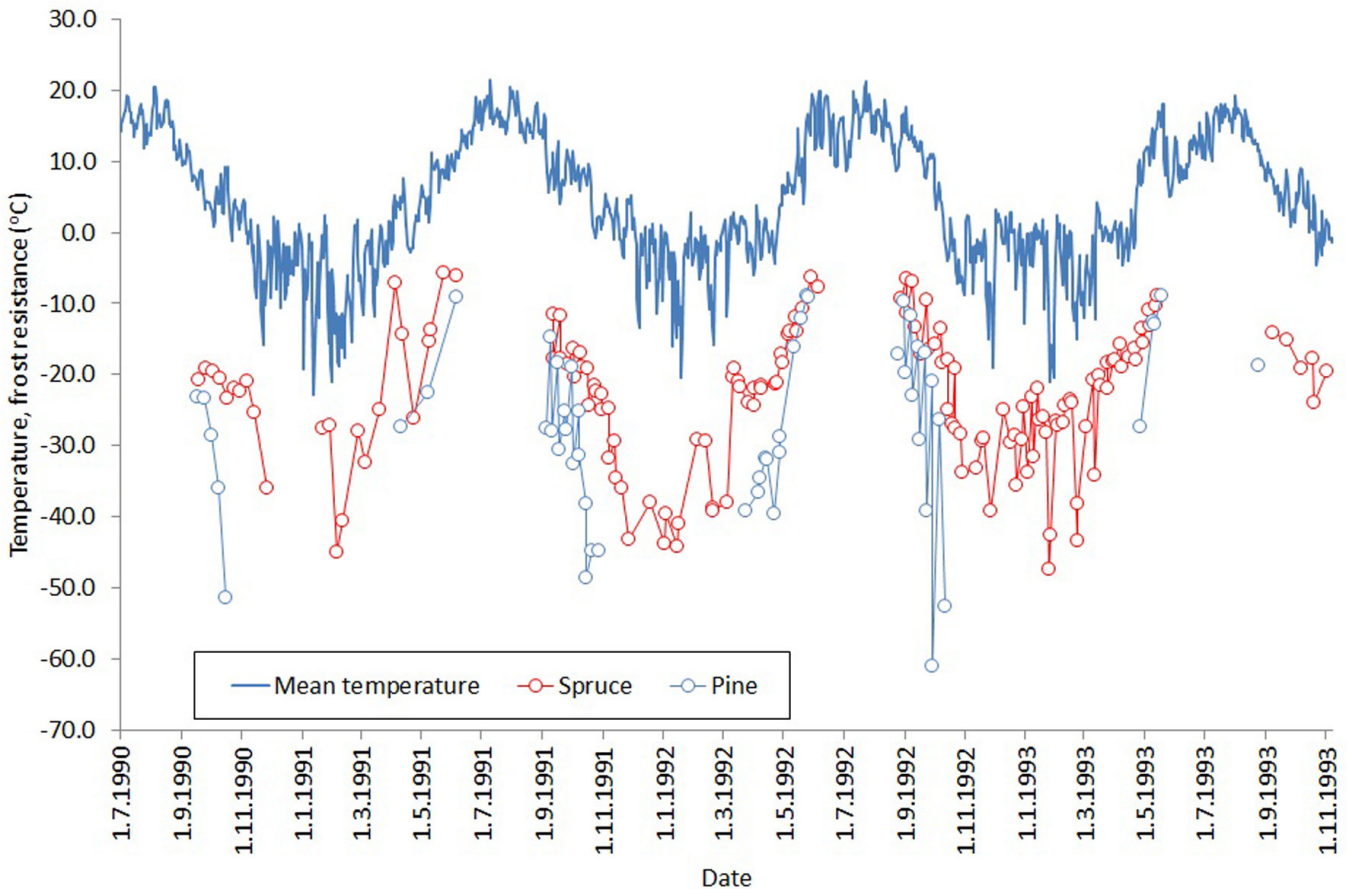
**Development of frost resistance of Norway spruce (red) and Scots pine (blue) buds through three winters**. The solid line shows the daily mean temperature for the study period. The figure is a combination of Figures 3, 6 in Beuker et al. (1998) (reproduced with permission), who tested frost resistance by exposing seedlings to freezing temperatures and measuring the subsequent leakage of electrolytes.

During the mild winter period, our spruce trees exhibited considerably higher values of fluorescence compared to pine. These results reflect those of Beuker et al. (1998), who tested frost tolerance by exposing seedlings to a range of freezing temperatures and measuring the subsequent leakage of electrolytes. They found that pine becomes frost tolerant much earlier during the autumn, loses it later in the spring, and tolerates lower temperatures during the winter (Figure 6, a reproduction of Figures 3, 6 in Beuker et al., 1998). Several experimental studies have shown that frost resistance and photosynthetic activity are linked (Leinonen, 1996; Repo et al., 2000, 2006; Mäkelä et al., 2004). Therefore, the conclusions of Beuker et al. (1998) are consistent with our results. We did not measure actual needle temperatures, as Martin et al. (1999) showed that needle temperatures of subalpine Pacific silver fir are typically ≤1°C higher than ambient, and we assumed a similar relationship was applicable in southern Finland.

In this study, the crossover parameter values (Table 3, Figure 3) were lower in the spring (i.e., 2012 needles) compared to the autumn (i.e., 2013 needles) for both species. This indicates a lower activity of light reactions at any temperature in the spring compared to the autumn. This agrees with Repo et al. (2006), who noted that the temperature-linked photosynthetic activity is different in autumn and spring and that photosynthetic potential is higher in autumn compared to spring, when estimated with light-saturated assimilation rate and the apparent quantum yield achieved with gas exchange measurements.

Results presented here indicate that Norway spruce regains the potential for photosynthetic activity more rapidly than Scots pine when temperatures suddenly warm during the cold months. The results disagree with those of Lundmark et al. (1988), who suggested these species to be similar in this respect. Norway spruce is considered to be more prone to spring frost damage due to a preference for frost-prone sites and sensitivity of new shoots (Lundmark and Hällgren, 1987). Our results indicate that this sensitivity is also a consequence of earlier reactivation of photosynthesis.

Because the Scots pine in our study had a more northerly origin than the Norway spruce, we expected the pines to be better adapted to cold and that they would start photosynthesizing at lower temperatures (Hänninen et al., 2007). However, our results indicate that photosynthetic reactivation was actually more conservative in pines. We conclude that the difference between these two conifers is due to species-specific responses to a common environmental change rather than a consequence of sample tree provenance. Moreover, spruce trees were drawn from a wider area than the pines, so one would assume origin explains some of the variance in the model parameters for this species. However, we found no statistically significant relationship between tree origin and parameter values, and an explanation of the greater variation observed for spruce remains obscure.

We tested a range of time constant values from 1 to 12 days when calculating the moving average of temperature. In the end, the time constant had only a slight effect on model performance. The time constant, or response time, is believed to reflect the rate at which photosynthetic activity reacts to changes in the environment. Earlier studies have produced a wide range of response times, e.g., 2 days (Ottander and Öquist, 1991), 8 days (Kolari et al., 2007), or up to 12 days (Mäkelä et al., 2004). Our model fitted the data slightly better with a response time of 7 days, but with only slight differences in model performance between one value and another, it is difficult to select any specific value. We suspect that the flat distribution of model performance over a range of response times accounts for the wide range of values reported in the literature.

Differences in the phenology of photosynthesis between the two conifer species have important implications for modeling forest productivity and forestry planning in the future. According to our results, spruce reactivates the light reactions of its photosynthesis more easily. We speculate that this implies that spruce also more readily reactivates its photosynthesis, and is therefore better adapted to utilize the warmer spring temperatures. On the other hand, earlier onset of photosynthetic activity also runs a greater risk of frost damage, especially if variation in springtime daily temperatures increases. However, if daily temperature variation remains similar to that seen today, Norway spruce will gain an advantage in a warmer future climate. Our model of photosynthetic activity can be used in process-based growth/yield models to characterize the different patterns of photosynthesis and respiration for spruce and pine. It can also be used as a tool for exploring how the risk of frost will develop under future climate scenarios and weather patterns.

### Conflict of interest statement

The authors declare that the research was conducted in the absence of any commercial or financial relationships that could be construed as a potential conflict of interest.
